# Selenoprotein-P Deficiency Predicts Cardiovascular Disease and Death

**DOI:** 10.3390/nu11081852

**Published:** 2019-08-09

**Authors:** Lutz Schomburg, Marju Orho-Melander, Joachim Struck, Andreas Bergmann, Olle Melander

**Affiliations:** 1 Institut für Experimentelle Endokrinologie, Charité—Universitätsmedizin Berlin, corporate member of Freie Universität Berlin, Humboldt-Universität zu Berlin, and Berlin Institute of Health, D-13353 Berlin, Germany; 2 Department of Clinical Sciences, Malmö, Lund University, SE 214 28 Malmö, Sweden; 3 Sphingotec GmbH, Neuendorfstrasse 15A, D-16761 Hennigsdorf, Germany; 4 Department of Internal Medicine, Clinical Research Center, Skåne University Hospital, Jan Waldenströms gata 35, Bldg. 91, SE 214 28 Malmö, Sweden

**Keywords:** Selenoprotein-P, selenium, cardiovascular disease, prevention, supplementation

## Abstract

Selenoprotein-P (SELENOP) is the main carrier of selenium to target organs and reduces tissue oxidative stress both directly and by delivering selenium to protective selenoproteins. We tested if the plasma concentration of SELENOP predicts cardiovascular morbidity and mortality in the primary preventive setting. SELENOP was measured from the baseline exam in 2002–2006 of the Malmö Preventive Project, a population-based prospective cohort study, using a validated ELISA. Quintiles of SELENOP concentration were related to the risk of all-cause mortality, cardiovascular mortality, and a first cardiovascular event in 4366 subjects during a median (interquartile range) follow-up time of 9.3 (8.3–11) years using Cox proportional Hazards Model adjusting for cardiovascular risk factors. Compared to subjects in the lowest quintile of SELENOP, the risk of all three endpoints was significantly lower in quintiles 2–5. The risk (multivariate adjusted hazard ratio, 95% CI) decreased gradually with the lowest risk in quintile 4 for all-cause mortality (0.57, 0.48–0.69) (*p* < 0.001), cardiovascular mortality (0.52, 0.37–0.72) (*p* < 0.001), and first cardiovascular event (0.56, 0.44–0.71) (*p* < 0.001). The lower risk of a first cardiovascular event in quintiles 2–5 as compared to quintile 1 was significant for both coronary artery disease and stroke. We conclude that the 20% with lowest SELENOP concentrations in a North European population without history of cardiovascular disease have markedly increased risk of cardiovascular morbidity and mortality, and preventive selenium supplementation studies stratified for these subjects are warranted.

## 1. Introduction

Selenium (Se) is an essential micronutrient of fundamental importance for human health [1]. A small group of proteins contains selenium as the 21st proteinogenic amino acid selenocysteine in their primary sequence at the active site, e.g., the glutathione peroxidases, thioredoxin reductases, iodothyronine deiodinases, and others [2]. These so-called selenoproteins are encoded by a set of 25 genes in humans [3]. Inherited defects causing reduced selenoprotein biosynthesis lead to a complex disease syndrome with myopathic features, male infertility, abnormal thyroid hormones, and signs of increased oxidative stress associated with high ultraviolet light (UV) radiation sensitivity and eventually neuronal loss [4].

The trace element is supplied by a regular diet; however, selenium intake levels differ strongly around the world due to biogeochemical differences [5]. Accordingly, the Se status of different populations depends on their soil quality as well as on the origin and pattern of the diet. Most of Europe, Africa, and Asia are considered as insufficiently supplied in contrast to e.g., the United States or Canada, partly reflected in genetic adaptations in populations residing in very selenium poor areas [6].

A low selenium intake causes insufficient expression of selenoproteins, low Se concentrations in the circulation and tissues, as well as an increased risk for certain diseases including colorectal cancer [7], autoimmune thyroid disease [8] or a sub-responsive immune system [9]. Different biomarkers are available for Se status assessment, of which total serum or plasma selenium concentrations and selenoprotein P (SELENOP) levels are established and considered as most reliable [10,11]. Circulating SELENOP mainly derives from hepatocytes and serves a Se transport function. Target cells express SELENOP-receptors that belong to the family of lipoprotein receptor-related proteins and can thereby become preferentially supplied with the essential trace element under Se-deficient conditions [12]. In addition, SELENOP also shows enzymatic activity and may protect vascular endothelial cells from oxidative and nitrosative stress and damage [13].

However, SELENOP status has not been studied yet with respect to cardiovascular disease (CVD), and there is a lack of large prospective studies in the primary preventive setting for the potential relationship between SELENOP and mortality [14]. Given the role of SELENOP as functional marker of Se status and availability, and the need for a large enough group of healthy subjects with sub-optimal Se supply, we tested the association of SELENOP with cardiovascular disease in a large European prospective cohort study.

## 2. Materials and Methods

### 2.1. Study Population

The Malmö Preventive Project (MPP) is a Swedish single-center prospective population-based study. Between 1974–1992, in all 33,346 citizens of the city of Malmö in Southern Sweden were included. The recruited subjects were screened for traditional cardiovascular risk factors. Between 2002–2006, all subjects alive were invited for a re-examination in which 18,240 individuals participated. This re-examination in 2002–2006 forms the baseline exam of the current study. Here, cardiovascular risk factors were assessed, and plasma was separated and frozen to −80 degrees for later analyses. Approval was granted by the Regional Board of Ethics, Lund, Sweden (#2009/633). Of the 18,240 subjects, we excluded 2087 subjects who had had a cardiovascular disease event (coronary artery disease, myocardial infarction or stroke) prior to the baseline exam leaving 16,153 individuals. Of these, 15,743 had complete data on cardiovascular risk factors, from whom a random sample of 4500 subjects was selected for analysis of SELENOP, among whom 4366 had a stored EDTA-plasma available in which SELENOP was subsequently analyzed (Supplementary Figure S1).

All study subjects signed oral and written informed consent to participate and to publish the results, and the study protocols were approved by the Regional Board of Ethics in Lund, Sweden.

### 2.2. Clinical Examination and Assays

Participants underwent a medical history, physical examination, and laboratory assessment. Blood pressure was measured using an oscillometric device twice after 10 min of rest in the supine position. Diabetes mellitus was defined as fasting plasma glucose 7.0 mmol/L or above, a self-reported physician diagnosis of diabetes, or use of anti-diabetic medication. Cigarette smoking was elicited by a self-administered questionnaire, with current cigarette smoking defined as any use within the past year. Measurements of fasting serum total cholesterol, high density lipoprotein (HDL) cholesterol, and triglycerides were made according to standard procedures at the Department of Clinical Chemistry, Skåne University Hospital. low density lipoprotein (LDL) cholesterol was calculated according to Friedewald’s formula. Plasma SELENOP concentrations were measured in fasted ethylene-diamine-tetraacetic acid (EDTA)-plasma using a validated ELISA (selenOtest ELISA, selenOmed GmbH, Berlin, Germany) characterized recently in detail [15], which was independently proven as a highly reliable commercial assay [16]. Corresponding selenium concentrations were measured in a subsample of 284 subjects using total reflection X-ray fluorescence (Picofox S2, Bruker nano) as described [7].

### 2.3. Endpoints

The baseline plasma concentration of SELENOP was analyzed in the study sample of 4366 individuals free of any of the primary outcomes in question in relation to three primary outcomes: a first cardiovascular event, all-cause mortality and cardiovascular mortality (for event definitions, see below). In secondary analyses, we separated the cardiovascular disease endpoint into incidence of coronary artery disease (CAD) and stroke. The endpoints were retrieved through record linkage of the personal identification number of each Swedish individual and the Swedish Hospital Discharge Register (SHDR), the Swedish Cause of Death Register (SCDR), the Stroke in Malmö Register, and the Swedish Coronary Angiography and Angioplasty Registry (SCAAR). These registers were previously described and validated for classification of outcomes [17,18,19]. CAD was defined as fatal or nonfatal myocardial infarction, death due to ischemic heart disease, percutaneous coronary intervention (PCI), or coronary artery bypass grafting (CABG), whichever came first. Stroke was defined as fatal or nonfatal stroke. Follow-up for outcomes extended to 31 December 2014. Cardiovascular death was defined a main cause of death diagnosis, according to the death certificate, between codes 390–459 of the International Classification of Disease (ICD) version 9 or within the I-chapter of ICD version 10.

### 2.4. Statistics

We measured the concentration of SELENOP in plasma from the baseline examination (between 2002–2006) of 4366 subjects of the Malmö Preventive Project who were free from prior cardiovascular disease. Quintiles of SELENOP (lowest quintile defined as reference) were related to risk of (1) all-cause mortality, (2) cardiovascular mortality, and (3) a first cardiovascular disease event (fatal or non-fatal myocardial infarction or stroke, coronary revascularization or death due to coronary heart disease) during follow-up using Cox Proportional Hazards Models adjusted for age, gender, current smoking, systolic blood pressure, use of antihypertensive medication, diabetes mellitus, LDL-cholesterol, HDL-cholesterol, and body mass index. Correlation between plasma concentration of SELENOP and Se was tested using Spearman’s correlation. Statistical analysis was performed using SPSS (v22.0; IBM Corp., Armonk, NY, USA). A two-sided *p* < 0.05 was considered significant.

## 3. Results

Baseline characteristics of the study population, stratified for quintiles of baseline SELENOP plasma concentration, are shown in Table 1. The most evident difference according to baseline SELENOP quintile was a smoking prevalence of 28% in the lowest quintile of SELENOP as compared to 16–18% in the other four quintiles. Furthermore, there were slight but significant linear or non-linear differences between SELENOP quintiles for age, gender, diabetes mellitus, LDL-cholesterol, HDL-cholesterol, and body mass index (Table 1).

During a median (interquartile range) follow-up time of 9.3 (8.3–11) years, a total of 1111 deaths occurred. The largest number of deaths was observed in SELENOP quintile 1 (*n* = 314). The number of deaths decreased with higher quintiles and the lowest number of deaths was recorded in quintile 4 (*n* = 175), followed by a nominal increase in the number of deaths in quintile 5 (*n* = 215) (Table 2). In multivariate adjusted analyses, the risk of all-cause mortality was highly significantly lower in each of SELENOP quintiles 2–5 compared to the lowest SELENOP quintile with the lowest point estimate of the hazard ratio in quintile 4.

**Figure 1:**
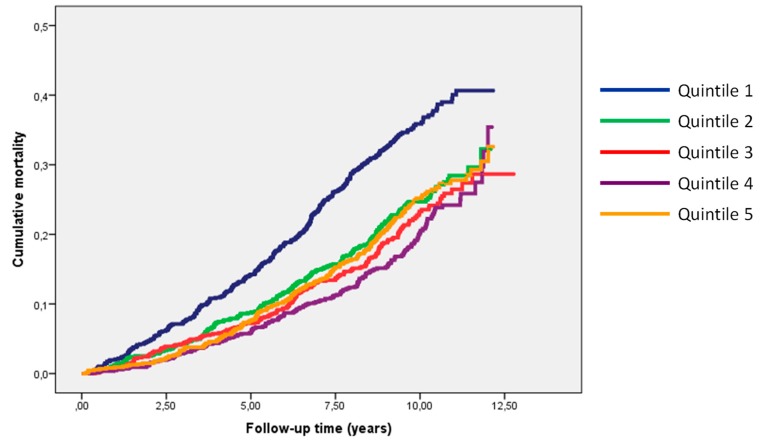
Kaplan Meier analysis for mortality risk in relation to Selenoprotein-P (SELENOP) status. Over the course of up to 12.5 years, the cumulative rates of mortality differed between the lowest quintile (Q1) of SELENOP plasma concentrations and the higher quintiles (Q2–Q5). A quantitative analysis is found in Table 2.

Similar patterns were observed for the crude and multivariate adjusted endpoint analyses of cardiovascular mortality (345 events) and risk of a first cardiovascular event (745 events), respectively, with significantly lower risks in each of SELENOP quintiles 2–5 compared to the bottom SELENOP quintile and the lowest point estimate of risk in SELENOP quintile 4 (Table 2). The individuals of each of SELENOP quintiles 2–5 had significantly lower risks as compared to the individuals of the lowest SELENOP quintile Q1 (Figure 1).

For this reason, we subsequently compared subjects of the lowest SELENOP quintile (SELENOP deficiency) with all subjects of SELENOP quintile 2–5 (normal SELENOP). In multivariate adjusted analyses, subjects with SELENOP deficiency as compared to subjects with normal SELENOP plasma concentration had a hazard ratio (95% confidence interval) of 1.51 (1.32–1.72) (*p* = 1.2 × 10^−9^) for all- cause mortality; 1.61 (1.32–2.09) (*p* = 1.7 × 10^−5^) for cardiovascular mortality, and 1.43 (1.21–1.69) (*p* = 2.7 × 10^−5^) for first cardiovascular event. When breaking up cardiovascular events into its two components, subjects with SELENOP deficiency were at significantly increased risk of both coronary artery disease (490 events) [1.27 (1.03–1.57) (*p* = 0.025)] and stroke (305 events) [1.57 (1.21–2.02) (*p* = 0.001)].

**Figure 2:**
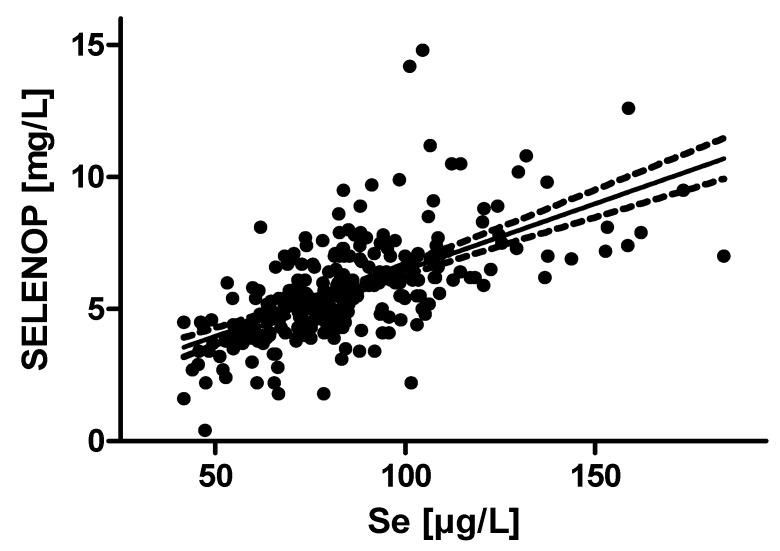
Correlation analysis between SELENOP and Se concentrations. A subset of 284 plasma samples was analyzed for both SELENOP and Se concentrations. The two biomarkers of Se status correlate strongly across the study cohort, indicative of sub-optimal Se intake (Spearman’s correlation coefficient; r = 0.6604).

In the subsample in which both SELENOP and selenium was measured there was significant correlation between the two parameters of Se status (R = 0.66, *p* = 4.0 × 10^−37^) (Figure 2).

Even though all analyses were adjusted for cardiovascular risk factors including smoking, we subsequently performed stratified analyses in smokers and non-smokers in order to make sure the elevated risk for cardiovascular morbidity and mortality in subjects with SELENOP deficiency was not caused by their higher smoking rates (Table 1).

Interestingly, among non-smokers SELENOP deficiency was significantly associated with all-cause mortality [1.56 (1.33–1.82) (*p* = 2.9 × 10^−8^)], cardiovascular mortality [1.88 (1.44–2.45) (*p* = 3.0 × 10^−6^)] and first cardiovascular event [1.47 (1.21–1.79) (*p* = 1.1 × 10^−4^)], whereas the association between SELENOP deficiency and the three main endpoints was weaker or non-significant among smokers (Table 3).

## 4. Discussion

We report a strong association between low SELENOP concentrations and the risk for all-cause mortality, cardiovascular mortality and a first cardiovascular event in a large group of adult Swedish subjects with no history of cardiovascular events prior to baseline, i.e., in a primary preventive setting. The low at-risk quintile (SELENOP Q1) identified is characterized by serum SELENOP concentrations below 4.3 mg/L SELENOP, corresponding to serum Se concentrations of less than 70 µg/L. These thresholds for the lowest quintile are similar to the corresponding values determined in the European prospective investigation of cancer and nutrition cohort (EPIC) [7]. Here, an analysis of 966 patients and 966 matched controls from eight different European countries identified an increased risk for colorectal cancer in the lowest quintiles of SELENOP and selenium concentrations, respectively, i.e., below a concentration of 3.6 mg/L of SELENOP or below 67.7 µg/L of total Se [7]. The slightly lower boundaries of SELENOP Q1 in the EPIC analysis may be related to the tendency that Northern European populations are better supplied with selenium than the subjects in central or southern parts of Europe [7].

A total serum selenium concentration in the range of 70 µg/L is known to indicate a sub-optimal expression of circulating selenoproteins including the glutathione peroxidases and SELENOP [10,20,21]. Full expression of SELENOP requires higher selenium intakes than that required for GPX1 or GPX3 saturation, and SELENOP is therefore considered as the most suitable protein-based biomarker of Se status becoming maximally expressed at serum or plasma selenium concentrations of 125 µg/L [10,20,21]. This concentration is found only in very few subjects of the population studied, and a linear association of plasma selenium concentrations with SELENOP levels is observed, indicating deficiency (Figure 2). In general, serum selenium concentrations of 125 µg/L or more are rarely observed in Europe, and a considerable fraction of the population is considered as selenium- deficient. The intake required for reaching a selenium status that might provide optimal protection from selenium-deficiency related diseases is unknown, but a U-shaped interaction between health risks or benefits and Se status is widely accepted [1,22,23]. Interestingly, Finland started a population-wide selenium supplementation effort more than 30 years ago and raised the average plasma selenium concentration from around 70 µg/L in 1985 to current levels of around 111 µg/L [24]. Our data suggest that this decision was most likely taken wisely, as hereby many subjects will have been promoted from the at risk quintile Q1 determined in this study to a higher SELENOP status. Yet, our study is reporting associations only, and should not be mis-interpreted as proof of causality.

Our results contribute a novel aspect to the abundant literature on selenium status and cardiovascular disease [25]. Specifically, we provide evidence on the potential relevance of the selenium transporter SELENOP in relation to cardiovascular morbidity and mortality. SELENOP may modify cardiovascular disease risk by several mechanisms [26]: It transports selenium to vital tissues that are equipped with receptors (megalin or APOER2) for SELENOP uptake, thereby increasing intracellular selenoprotein biosynthesis for improving antioxidative defense and protein quality control systems [12]. SELENOP exhibits GPX activity and is capable of catalyzing degradation of phospholipid hydroperoxides, thereby protecting cell membrane integrity [27] and LDL-particles from oxidation [28]. SELENOP is also known to reducing peroxynitrite [29], and to associate with the extracellular matrix via a heparin binding domain [30]. Finally SELENOP binds heavy metals like Cd, As, and Hg thereby reducing oxidative stress and avoiding toxic damage in the circulation [31]. Especially the latter notion has been supported by a recent study with Hg-exposed Inuit, where subjects with high selenium intake and status were less hypertensive and displayed reduced stroke and myocardial infarction rates as compared to those with a lower selenium status [32].

Our results align with prior studies on the inverse relation of certain selenoproteins with cardiovascular disease risk. In a prospective study of >600 patients with suspected coronary artery disease, GPX1 erythrocyte activity was related to the risk of cardiovascular events, independent from smoking status [33]. Similarly, circulating levels of the extracellular GPX isoform (GPX3) were inversely related to the risk of cardiovascular events in patients with atrial fibrillation in a prospective cohort study with 909 patients [34]. Notably, both studies had been performed in countries with insufficient selenium intake, i.e., Germany and Italy, respectively. The recent data from the Minnesota Heart Survey also indicate that selenium status in the form of GPX3 activity is inversely correlated to cardiovascular disease mortality even in a selenium replete population [35]. GPX3 is a valid biomarker for chronic kidney disease, contributing to overall selenium status and affecting systemic oxidative stress [36]. Notably, renal GPX3 expression depends on liver-derived SELENOP [37], and SELENOP should thus be considered as a more direct and reliable biomarker of selenium status [38].

While an inverse relation between selenium status and cardiovascular disease risk is found in most of the clinical studies, the results from intervention trials are ambiguous [39]. A recent meta- analysis showed that selenium supplementation does not generally reduce cardiovascular disease risk, probably due to the inclusion of results from studies conducted in areas with relatively high baseline selenium status without selecting individuals with low selenium status [40].

**Figure 3:**
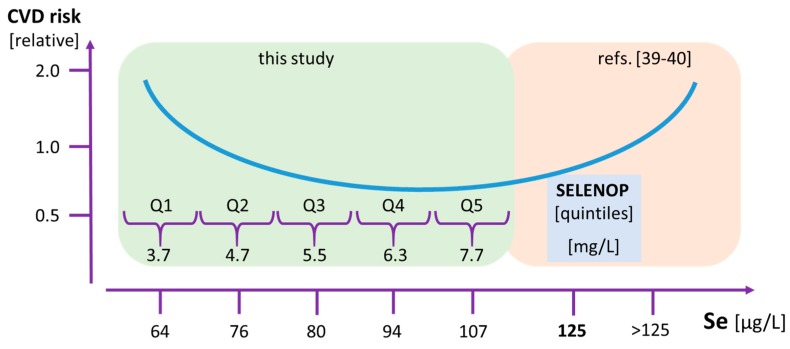
Presumed U-shaped interaction of Se status and CVD risk. The present study indicates a highly increased risk for cardiovascular endpoints in subjects residing in the lowest quintile (Q1) of SELENOP status as compared to the other subjects with higher SELENOP status (Q2–Q5). The figure presents both the plasma SELENOP and corresponding total Se concentrations. The green shaded area denotes the results from the current study, whereas the red shaded part presents an extrapolation of published studies from areas with higher baseline selenium status.

In combination with the findings from selenium-replete subjects where a positive interaction of very high selenium status with hypertension has been observed [41], and lowest mortality risk is seen in subjects with lowest Se levels [42], our data reinforce the idea of a U-shaped interaction between selenium status and mortality risk. Specifically, our study highlights the lower boundary of selenium intake and selenium status (Figure 3). The cardiovascular disease and mortality risks of the majority of our study subjects were independent of the selenium status, indicating that their selenium status was within the plateau phase connecting selenium-deficiency from selenium-oversupply. However, about 20% of subjects, i.e., the ones residing in the lowest quintile Q1, exhibited a strongly increased health risk and may profit from supplemental selenium.

This notion has several potential clinical implications: (1) Subjects with potentially low selenium intake should be tested for SELENOP deficiency and advised with respect to taking natural selenium rich products or supplements. (2) There is a need for randomized controlled trials (RCT) in selenium-deficient populations specifically in subjects with SELENOP concentrations corresponding to SELENOP Q1, to verify that selenium-containing supplements or a selenium-rich diet can increase SELENOP levels and thereby reduce cardiovascular disease risk in these subjects. (3) Natural, environmental and pharmacological modifiers of SELENOP expression need to be identified in order to better control selenium status and be tested in relation to cardiovascular disease risk. Our study has limitations. Due to the observational nature of the study, we cannot prove that the associations between SELENOP and the study endpoints are causal. For this, RCTs targeting the low SELENOP segment of the population are needed. Moreover, the MPP included more men than women and our study population, surveyed 2002–2006, represents survivors from the original baseline examination 1974–1992 and thus the subjects enrolled are likely healthier than the background population.

## 5. Conclusions

We conclude that in a North European population without history of cardiovascular disease, the 20% with lowest SELENOP concentrations have markedly increased risk of cardiovascular morbidity and mortality. Rather than population-wide supplementation strategies, clinical trials testing if cardiovascular morbidity and mortality can be reduced in subjects belonging to this low SELENOP stratum, are warranted.

## 6. Patents

Dr. Bergmann reports being president of Sphingotec GmbH, which holds the patent rights for the use of Selenoprotein-P in prediction of cardiovascular disease. Prof. Melander reports being listed as inventor on the same patent application.

## Figures and Tables

**Table 1:** Baseline clinical characteristics according to quintile (Q) of concentration of Selenoprotein-P (SELENOP) at baseline of the subjects analyzed who were without history of cardiovascular disease.

	Q1	Q2	Q3	Q4	Q5	*p*	*p*-Trend
	(*n* = 873)	(*n* = 873)	(*n* = 874)	(*n* = 873)	(*n* = 873)		
SELENOP ^1^ (mg/L)	3.7 (0.4–4.3)	4.7 (4.3–5.1)	5.5 (5.1–5.9)	6.3 (5.9–6.9)	7.7 (6.9–20)	n. a.	n. a.
Age (years)	70 ± 6.4	69 ± 6.4	69 ± 6.1	69 ± 6.1	70 ± 6.1	<0.001	0.01
Gender, *n* (%) male	594 (68)	641 (73)	587 (67)	633 (73)	553 (63)	<0.001	0.04
Current smoking, *n* (%)	240 (28)	157 (18)	149 (17)	146 (17)	143 (16)	<0.001	<0.001
Systolic blood pressure (mmHg)	147 ± 21	147 ± 20	146 ± 21	146 ± 20	147 ± 20	n. s.	n. s.
Antihypertensive medication, *n* (%)	311 (36)	277 (32)	321 (37)	289 (33)	278 (32)	n. s.	n. s.
Diabetes Mellitus, *n* (%)	86 (9.9)	83 (9.5)	79 (9.0)	103 (12)	115 (13)	0.024	0.007
LDL-cholesterol (mmol/L)	3.62 ± 0.98	3.73 ± 0.96	3.74 ± 0.93	3.74 ± 0.97	3.71 ± 0.99	0.043	n. s.
HDL-cholesterol (mmol/L)	1.37 ± 0.42	1.34 ± 0.39	1.36 ± 0.38	1.39 ± 0.39	1.44 ± 0.43	<0.001	<0.001
Body Mass Index (kg/m^2^)	26.9 ± 4.6	27.3 ± 4.2	27.5 ± 4.3	27.0 ± 3.8	27.0 ± 4.1	0.034	n. s.

^1^ SELENOP; plasma concentration of selenoprotein *P*, Q; quintile, LDL; low density lipoprotein, HDL; high density lipoprotein, n. a.; not applicable, n. s.; non-significant.

**Table 2:** Population quintile (Q) of SELENOP in relation to all-cause mortality, cardiovascular mortality and a first cardiovascular event in subjects without history of cardiovascular disease at baseline in multivariate adjusted models.

Parameter	Q1	Q2	Q3	Q4	Q5
	(*n* = 873)	(*n* = 873)	(*n* = 874)	(*n* = 873)	(*n* = 873)
SELENOP ^1^ (mg/L)	3.7	4.7	5.5	6.3	7.7
	(0.4–4.3)	(4.3–5.1)	(5.1–5.9)	(5.9–6.9)	(6.9–20)
ALL-CAUSE MORTALITY					
Number of events	314	214	193	175	215
Hazard Ratio (95% CI)	1.0	0.73 ***	0.66 ***	0.57 ***	0.69 ***
	(ref)	(0.61–0.87)	(0.55–0.79)	(0.48–0.69)	(0.58–0.82)
CARDIOVASCULAR MORTALITY					
Number of events	106	66	66	53	60
Hazard Ratio (95% CI)	1.0	0.65 **	0.66 **	0.52 ***	0.59 **
	(ref)	(0.48–0.89)	(0.48–0.89)	(0.37–0.72)	(0.43–0.81)
FIRST CARDIOVASCULAR EVENT					
Number of events	188	157	145	115	140
Hazard Ratio (95% CI)	1.0	0.79 *	0.75 *	0.56 ***	0.70 **
	(ref)	(0.64–0.98)	(0.61–0.94)	(0.44–0.71)	(0.56–0.87)

^1^ SELENOP; plasma concentration of selenoprotein P; CI, confidence interval. All analyses were adjusted for age, gender, current smoking, systolic blood pressure, use of antihypertensive medication, diabetes mellitus, LDL-cholesterol, HDL-cholesterol, and body mass index. * *p* < 0.05; ** *p* < 0.01; *** *p* < 0.001.

**Table 3:** Association of SELENOP status in relation to major endpoints in non-smokers vs. smokers.

Parameter	Non-Smokers (*n* = 3531)		Smokers (*n* = 835)	
SELENOP-Deficient vs. Normal	Hazard Ratio (95% CI)	*p*-Value	Hazard Ratio (95% CI)	*p*-Value
All-cause mortality	1.56 (1.33–1.82)	<0.001	1.35 (1.05–1.74)	0.018
CVD mortality	1.88 (1.44–2.45)	<0.001	1.23 (0.70–1.80)	NS
First CVD event	1.47 (1.21–1.79)	<0.001	1.32 (0.95–1.82)	NS

CVD, cardiovascular disease.

## Supplementary Materials

The following are available online at https://www.mdpi.com/2072-6643/11/8/1852/s1, Figure S1: Consort Diagram.
