# Supplementary figures and images for: Selenoprotein-P Deficiency Predicts Cardiovascular Disease and Death
Source: Nutrients. 2019 Aug 9;11(8):1852. doi: 10.3390/nu11081852 (PMC6723215; doi:10.3390/nu11081852)

Supplementary Figure S1: Consort Diagram

CONSORT DIAGRAM

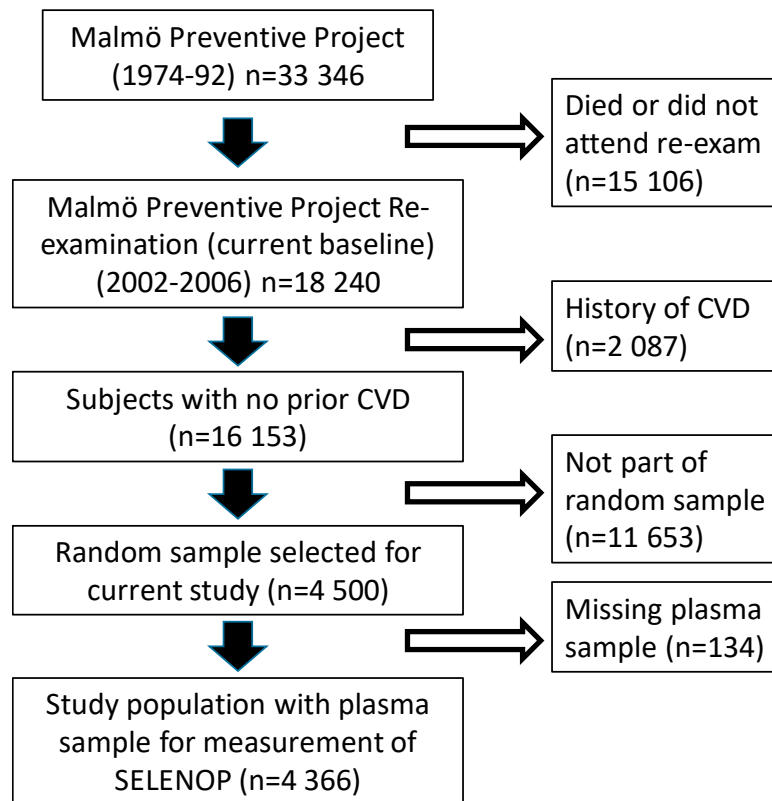

Supplement: Supplementary file 1 [file nutrients-11-01852-s001.pdf]
